# Red blood cell distribution width is not correlated with preeclampsia among pregnant Sudanese women

**DOI:** 10.1186/1746-1596-9-29

**Published:** 2014-02-05

**Authors:** Hala Abdullahi, Ameer Osman, Duria A Rayis, Gasim I Gasim, Abdulmutalab M Imam, Ishag Adam

**Affiliations:** 1 Department of Obstetrics and Gynecology, Faculty of Medicine, University of Khartoum, Khartoum, Sudan; 2 College of Medicine, Qassim University, Qassim, Saudi Arabia

**Keywords:** Preeclampsia, Red cell distribution width, Pregnancy Sudan

## Abstract

**Background:**

Preeclampsia is a leading cause of maternal and perinatal mortality worldwide. The exact etiology of preeclampsia is unknown, but the inflammatory process is postulated as one of the etiologies. Red blood cell distribution width (RDW) is a measure of anisocytosis (variation of red cell size) and is associated with hypertension and diabetic ketoacidosis. There are few data on the association between RDW and preeclampsia. This study aimed to investigate the association between RDW and preeclampsia.

**Methods:**

A case–control study was conducted at Khartoum Hospital, Sudan, during June to August 2012. Cases were women with preeclampsia and healthy women were controls. Sociodemographic characteristics, obstetrics, and clinical data were recorded. The complete blood count, including RDW, was measured using an automated hematology analyzer.

**Results:**

The cases and controls (65 women in each arm) were matched in their basic characteristics. There was no difference in the mean (SD) RDW between women with preeclampsia and controls (14.5 ± 1.8% vs. 14.4 ± 1.4%, P = 0.710). There was also no difference in the mean RDW between women with mild and severe preeclampsia (14.7 ± 1.9% vs. 13.9 ± 1.4%, P = 0.144. In logistic regression, there was no association between RDW and preeclampsia (OR = 0.9, CI = 0.7–1.1, P = 0.952).

**Conclusions:**

RDW levels are not associated with the presence or severity of preeclampsia.

**Virtual slides:**

The virtual slide(s) for this article can be found here: http://www.diagnosticpathology.diagnomx.eu/vs/1206247718115175

## Background

Preeclampsia is a leading cause of maternal and perinatal mortality worldwide [1]. The exact underlying pathology for the development of preeclampsia is not yet completely understood. Placental pathology causing preeclampsia has been suggested, with inadequate cytotrophoblast invasion and widespread maternal endothelial dysfunction [2]. Increased erythropoietic stimulation associated with underlying placental hypoxia has been observed in preeclampsia [3].

Red cell distribution width (RDW) is a parameter, which is usually evaluated in a fully automated hematology analyzer, as part of the complete blood count. RDW is a marker of anisocytosis (red cell size variation). RDW can reflect early changes in red blood cells, and these are accompanied by iron deficiency anemia, and have been shown to have a high sensitivity at detecting anemia [4,5].

Recent research has shown that RDW is higher in pre-hypertension and hypertension in the non-pregnant population [6]. Furthermore, highly significant associations have been described between RDW values and diabetic ketoacidosis, cardiovascular and thrombotic disorders, and cardiac mortality in patients with coronary artery disease, acute and chronic heart failure, and stroke [7,8]. However, the vast majority of the data on RDW and hypertension are on the non-pregnant population, with few data on RDW and preeclampsia [9].

Therefore, the current study was conducted to investigate the association between RDW and preeclampsia at Khartoum Hospital, Sudan. This study aimed to add to the knowledge on pathogenesis on preeclampsia [10-13] and examine the diagnostic value of RDW in various diseases in this setting (Sudan) [14]. In Sudan, there is an extremely high maternal mortality, with preeclampsia/eclampsia accounting for 4.2% of obstetric complications and 18.1% of maternal deaths [15,16].

## Methods

A case–control study was carried out at Khartoum Hospital, Sudan, during June to August 2012. Khartoum Hospital is a tertiary hospital serving the local community and receiving medical referrals from other parts of Sudan. After signing an informed consent, women carrying a singleton pregnancy were enrolled. A case was defined as a woman with preeclampsia who was diagnosed according to the criteria of the international society for the study of hypertension in pregnancy [17]: a diastolic blood pressure of ≥90 mmHg measured 4 hours apart, plus proteinuria of more than 1+ by dipsticks or a 24-hour urine level ≥300 mg. Cases were further divided into mild and severe preeclampsia according to diastolic blood pressure <110 or ≥110 mmHg. Pregnant women with multiple pregnancies or medical disorders, such as diabetes mellitus or inflammatory conditions, were excluded. The control group included healthy pregnant women with no known medical or obstetric complications. A pre-tested questionnaire was used to gather data from each woman in cases and control groups. The women’s age, parity, and gestational age were recorded. Weight and height were measured, and body mass index (BMI) was calculated as weight in kilograms divided by the square of height in meters. The maternal complete blood count was measured for all women using an automated hematology analyzer as previously described [14,18,19].

A sample size of 65 subjects in each arm of the study was calculated using the OpenEpi-Epidemiological calculator with 80% power and a confidence interval of 95% to detect a difference of 5% at α = 0.05, with 10% of non-respondents/incomplete data.

Ethical approval for the study was obtained from the Khartoum Hospital Ethical Board.

### Statistics

Data were analyzed using SPSS version 20 for Windows (SPSS Inc., Chicago, IL, USA). Mean (SD) and proportions of the variables were compared using the Student’s t-test and χ^2^ test, respectively. A P value <0.05 was considered significant. Logistic regression analysis was used to assess the factors associated with preeclampsia as the dependent factor, and age, parity, RDW, platelets, and WBC count as independent factors.

## Results

A total of 130 women (65 in each arm) were enrolled in the study. The two groups were well matched in their basic characteristics, with no significant differences in mean age, parity, and BMI. There were also no differences in mean hemoglobin concentration, platelet count, and white cell count between the case and control groups (Table 1).

**Table 1:** The mean (SD) of the characteristics of women with preeclampsia and controls

**Variables**	**Women with preeclampsia**	**Controls**	**P**
	**(n = 65)**	**(n = 65)**	
Age, years	29.6 (5.4)	29.1(4.6)	0.604
Parity	2.8(1.8)	2.7(1.9)	0.882
Body mass index kg/^2^ cm	23.8(2.1)	24.2(1.8)	0.245
Hemoglobin, g/dl	11.4(1.4)	11.7(1.2)	0.192
Platelets × 10^3^ μL	220153(8477)	221752(6899)	0.906
White blood cell × 10^3^ μL	7489 (3308)	8000(2204)	0.906

There were no differences in age, parity, BMI, hemoglobin concentration, platelet count, and white blood cells between women with mild preeclampsia and those with severe. However, the platelet count was significantly lower in women with severe preeclampsia than in those with mild preeclampsia (P < 0.001, Table 2).

**Table 2:** The mean (SD) of the of characteristics of women with mild and severe preeclampsia

**Variables**	**Women with mild preeclampsia (n = 15)**	**Women with severe preeclampsia (n = 50)**	**P**
Age, years	29.8 (5.1)	28.9 (5.2)	0.563
Parity	2.7 (1.5)	3.3 (2.4)	0.238
Body mass index kg/^2^ cm	23.6 (2.6)	24.4 (1.8)	0.195
Hemoglobin, g/dl	10.4 (1.3)	10.7 (1.5)	0.760
Platelets × 10^3^ μL	243760 (8141)	141466 (31999)	<0.001
White blood cell × 10^3^ μL	7388 (4089)	7826 (4089)	0.657

**Figure 1:**
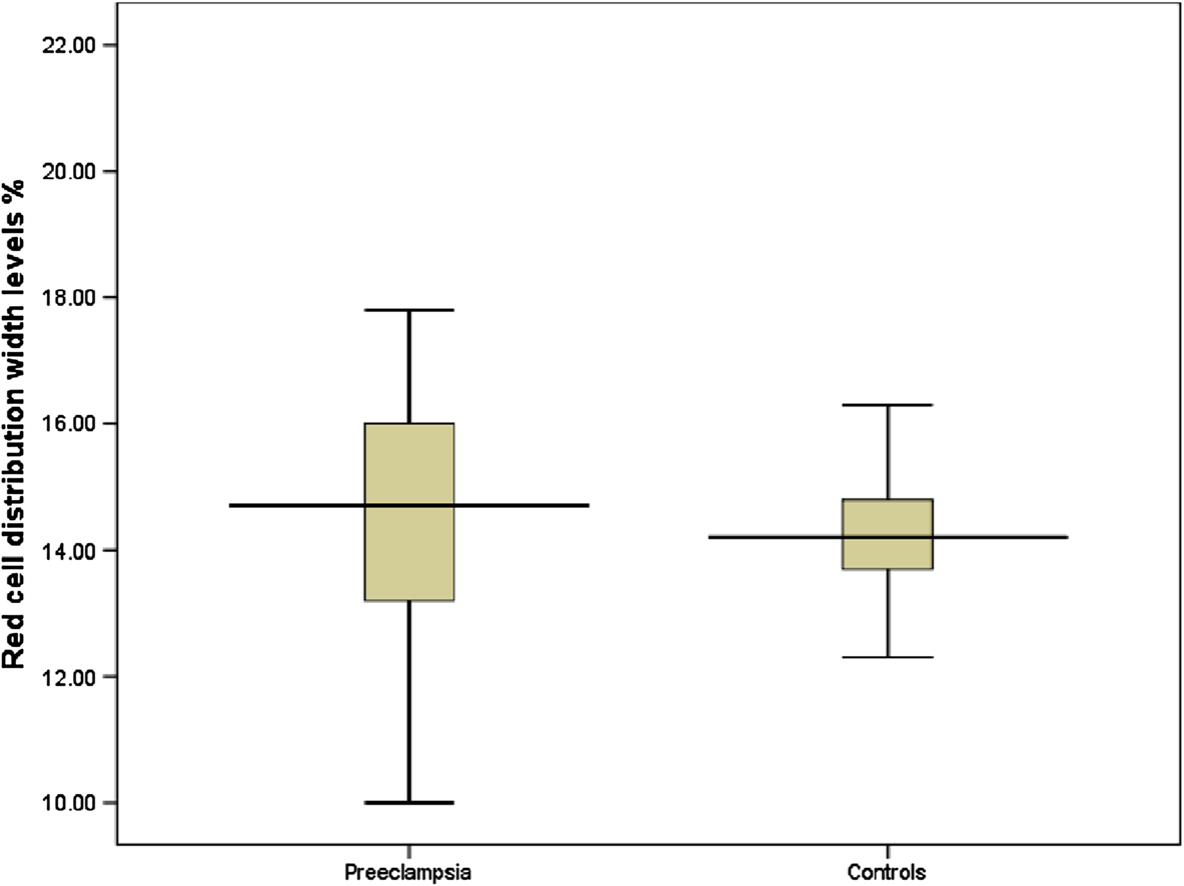
Comparison of red cell distribution width levels in the preeclampsia and control groups.

**Figure 2:**
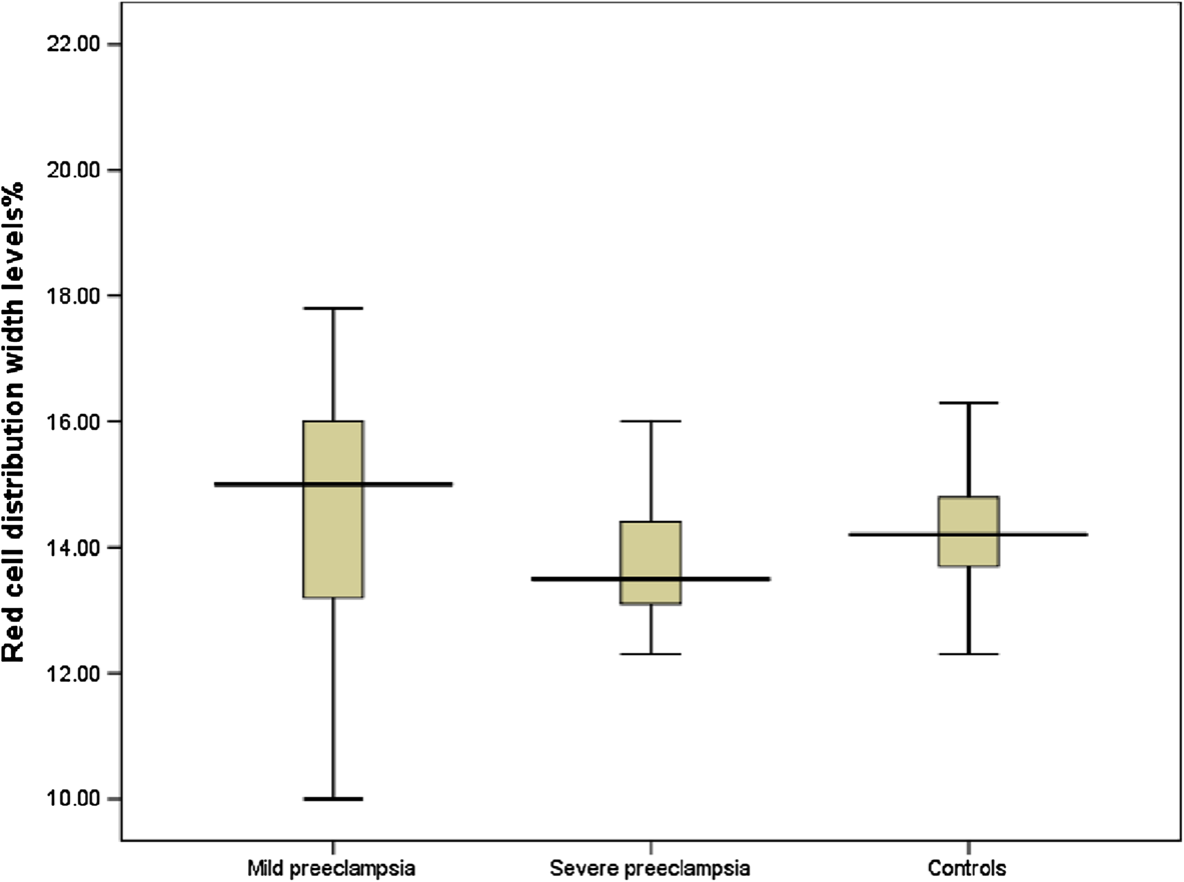
Comparison of red cell distribution width levels in the preeclampsia and control groups.

There was no difference in the mean (SD) RDW between women with preeclampsia and controls 14.5 ± 1.8% vs. 14.4 ± 1.4%, P = 0.710, Figure 1). There was no difference in the mean RDW between women with mild and severe preeclampsia 14.7 ± 1.9% vs. 13.9 ± 1.4%, P = 0.144, Figure 2).

**Figure 3:**
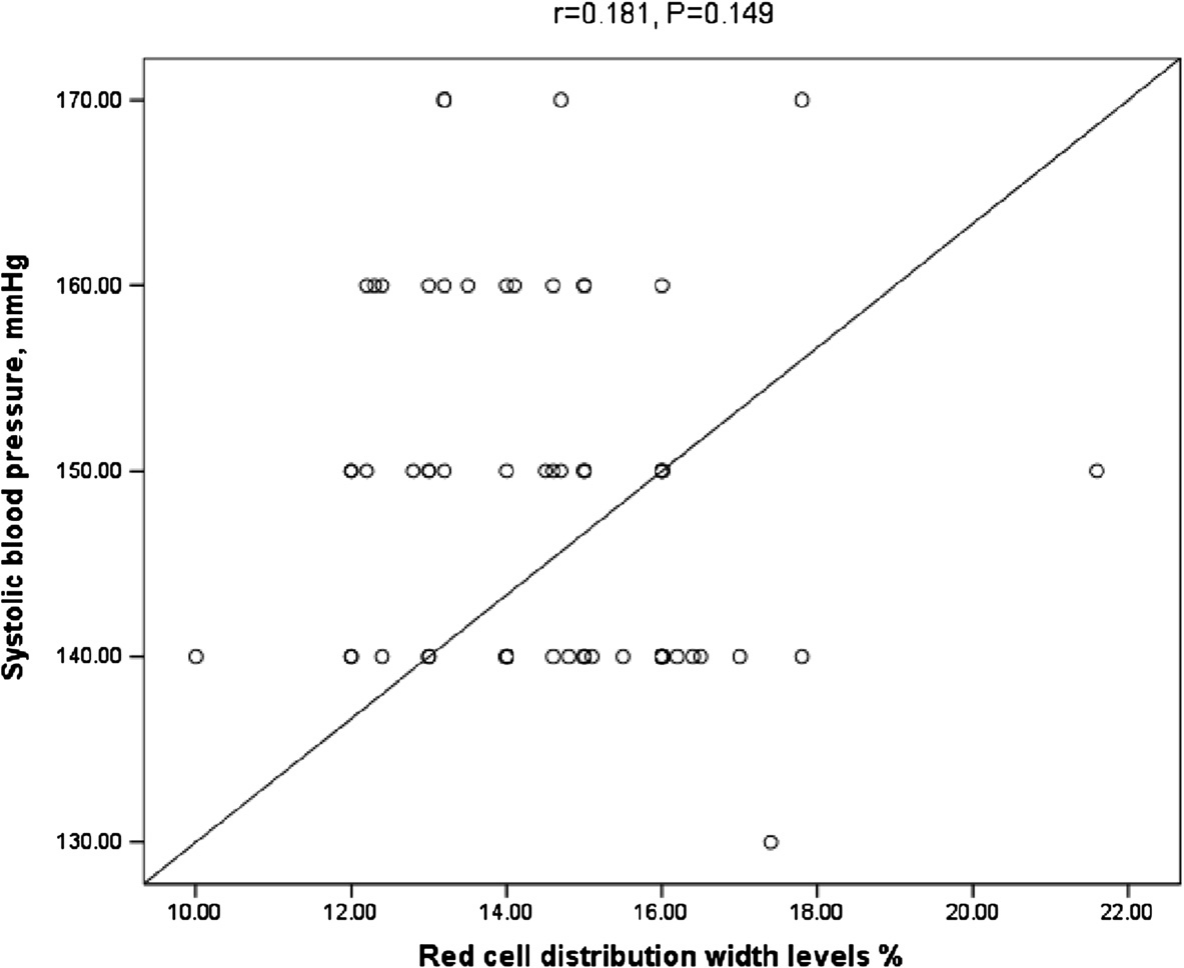
Correlation between RDW and systolic blood pressure.

In logistic regression, there was no association between RDW and preeclampsia (odds ratio = 0.9, confidence interval = 0.7–1.1, P = 0.952, Table 3).There was no correlation between RDW and systolic blood pressure, Figure 3.

**Table 3:** Factors associated with preeclampsia using univariate and multivariate analyses

	**Univariate analyses**	**Multivariate analyses**
**Variable**	**OR 95% CI P**	**OR 95% CI P**
Age	0.9 0.9–1.0 0.601	0.9 0.9–1.0 0.468
parity	0.9 0.8–1.1 0.881	1.0 0.8–1.2 0.959
Platelets	1.0 0.9–1.0 0.905	1.0 0.9– 1.0 0.938
Red cell distribution width	0.9 0.7–1.2 0.961	0.9 0.7–1.1 0.952
White blood cells	1.0 0.9 –1.2 0.942	1.0 0.9–1.2 0.262

OR = Odds ratio, CI = confidence interval.

## Discussion

The current study showed no difference in RDW between women with preeclampsia and controls. There was also no difference in RDW between women with mild and severe preeclampsia. In addition, in the current study, there was no correlation between RDW and preeclampsia.

Recently, Keskin and colleagues observed that RDW was significantly higher in the women with preeclampsia and RDW was associated with the severity of preeclampsia [9]. There are few data on the influence of normal pregnancy on RDW, but a high RDW between 34 weeks of gestation and the onset of labor has been reported [20]. RDW has been recently observed to be associated with hypertension and it is an indicator of poor prognosis in acute myocardial infarction and heart failure [6,21-23]. Although the mechanism of the relationship between RDW and hypertension is not clearly understood, increased inflammation is the most plausible theory [24]. Interestingly, the above mentioned increased inflammation theory of RDW and hypertension has recently been supported by a positive correlation between C reactive protein and RDW levels in preeclamptic women [9]. Inflammation might increase RDW levels via impairment of iron metabolism and disruption of the response to erythropoietin. This could cause immature erythrocytes to enter the circulation by impairing erythrocyte maturation [25,26].

Recently, a variety of studies have supported that RDW might be a useful parameter for gathering useful information, either diagnostic or prognostic, in different diseases [6-8]. However, it is still unclear whether anisocytosis (reflected by RDW) might be the cause, or a simple epiphenomenon of an underlying disease, or perhaps an element of both. Nevertheless, RDW is an easy, inexpensive, routinely reported investigation, which might allow the acquisition of significant diagnostic and prognostic information in patients with hypertension and preeclampsia.

The limitations of the current study are that iron, vitamins (including folic acid), and trace elements were not investigated. We have previously shown a high level of deficiency in these elements among pregnant Sudanese women [13,27], and their deficiency might be responsible for preeclampsia or RDW. Furthermore the previous finding of ascoaition between severe anemia and preeclampsia might need further investigation [11].

## Conclusion

In this study we show that RDW levels are not associated with the presence or severity of preeclampsia.

## Competing interests

The authors declare that they have no competing interests.

## Authors' contributions

HA and IA designed the study. DAR and GIG conducted the clinical part of the study. AMI and GIG participated in the laboratory work. AMI and IA performed statistical analysis. All of the authors approved the final version of the manuscript.
